# Supplementary material for: An open-source framework for large-scale, flexible evaluation of biomedical text mining systems
Source: J Biomed Discov Collab. 2008 Jan 29;3:1. doi: 10.1186/1747-5333-3-1 (PMC2276192; doi:10.1186/1747-5333-3-1)
Supplement: Additional file 1 — Evaluation platform source code. The additional file contains the software evaluation framework discussed in this paper. The most current release can be downloaded from SourceForge.net [1]. See the accompanying README file for instructions on installation and use. Also included in the distribution is a collection of UIMA wrappers for some commonly used BioNLP tools and annotated corpora. [file 1747-5333-3-1-S1.zip › bionlp_uima_repository-1.0.1/docs/api/edu/uchsc/ccp/util/nlp/annotation/comparison/EntityPartsAnnotationComparator.html]

EntityPartsAnnotationComparator (Center for Computational Pharmacology Util/UIMA API)


|  |  |  |
| --- | --- | --- |
| **Overview** \| **Package** \| **Class** \| **Tree** \| **Deprecated** \| **Index** \| **Help** | |  |
| **PREV CLASS**   **NEXT CLASS** | **FRAMES**    **NO FRAMES**     **All Classes** |  |
| SUMMARY: NESTED \| FIELD \| CONSTR \| METHOD | DETAIL: FIELD \| CONSTR \| METHOD |  |


---


## edu.uchsc.ccp.util.nlp.annotation.comparison Class EntityPartsAnnotationComparator

```
java.lang.Object
  edu.uchsc.ccp.util.nlp.annotation.comparison.AnnotationComparator
      edu.uchsc.ccp.util.nlp.annotation.comparison.EntityPartsAnnotationComparator
```

**All Implemented Interfaces:**: Comparator<TextAnnotation>

---

``` public class EntityPartsAnnotationComparator extends AnnotationComparator ```

This class implements the functionality of the "Protein Name Parts" comparison metric described in Olson et al...  
The usefulness of this kind of comparison is limited to simple entity mentions, therefore, the mention comparison will compare the entire mention
hierarchy, however, the entity parts computation will only take place on the base annotation span.  
  
For example, if for some reason you wanted to run the entity-parts metric on a group of transport annotations, where each transport mention has
complex slot mentions (transported entities, source, destination, transport participants), then this method will  

1. find equivalent transport annotations using the SloppySpanComparator- if equivalent annotations are found, then the covered text for each annotation (and in this case, just for the transport annotation, not any
     of its slot fillers) will be tokenized on whitespace, and the entity-parts metric will be done using those tokens.

**Author:**
:   williamb

---

**Constructor Summary**

`EntityPartsAnnotationComparator()`


| **Method Summary** | |
| --- | --- |
| `PRFResult` | `compare(List<TextAnnotation> goldTaList, List<TextAnnotation> testTaList)`             Default comparison of two lists of TextAnnotations utilizing the StrictSpanComparator and the IdenticalMentionComparator family |
| `PRFResult` | `compare(List<TextAnnotation> goldTaList, List<TextAnnotation> taList, SpanComparator spanComparator, ClassMentionComparator classMentionComparator)`             Overridden - note this ignores all extraneous input parameters and simple returns EntityPartsAnnotationComparator.compare(List goldTaList, List testTaList) |
| `PRFResult` | `compare(List<TextAnnotation> goldTaList, List<TextAnnotation> testTaList, SpanComparator spanComparator, ClassMentionComparator classMentionComparator, int maximumComparisonDepth)`             Overridden - note this ignores all extraneous input parameters and simple returns EntityPartsAnnotationComparator.compare(List goldTaList, List testTaList) |
| `int` | `compare(TextAnnotation goldTA, TextAnnotation testTA)`             Since this method returns an integer, it does not make sense to use it to compare the entity parts, however, in order to override the default compare() method of AnnotationComparator which employs the strict span comparator, we will implement this method to return 0 if the resulting F measure is 1.0, -1 otherwise. |
| `int` | `compare(TextAnnotation ta1, TextAnnotation ta2, SpanComparator spanComparator, ClassMentionComparator classMentionComparator)`             Overridden - note this ignores all extraneous input parameters and simple returns EntityPartsAnnotationComparator.compare(TextAnnotation ta1, TextAnnotation ta2) |
| `int` | `compare(TextAnnotation ta1, TextAnnotation ta2, SpanComparator spanComparator, ClassMentionComparator classMentionComparator, int maximumComparisonDepth)`             Overridden - note this ignores all extraneous input parameters and simple returns EntityPartsAnnotationComparator.compare(TextAnnotation ta1, TextAnnotation ta2) |

**Methods inherited from class edu.uchsc.ccp.util.nlp.annotation.comparison.AnnotationComparator**

`metaDataCompare`

**Methods inherited from class java.lang.Object**

`clone, equals, finalize, getClass, hashCode, notify, notifyAll, toString, wait, wait, wait`

**Methods inherited from interface java.util.Comparator**

`equals`

**Constructor Detail**

### EntityPartsAnnotationComparator

```
public EntityPartsAnnotationComparator()
```


**Method Detail**

### compare

```
public PRFResult compare(List<TextAnnotation> goldTaList,
                         List<TextAnnotation> testTaList)
```

:   **Description copied from class: `AnnotationComparator`**
:   Default comparison of two lists of TextAnnotations utilizing the StrictSpanComparator and the IdenticalMentionComparator family

    :   **Overrides:**: `compare` in class `AnnotationComparator`
    :   **Returns:**

---


### compare

```
public int compare(TextAnnotation goldTA,
                   TextAnnotation testTA)
```

:   Since this method returns an integer, it does not make sense to use it to compare the entity parts, however, in order to override the default
    compare() method of AnnotationComparator which employs the strict span comparator, we will implement this method to return 0 if the resulting F
    measure is 1.0, -1 otherwise.

    :   **Specified by:**: `compare` in interface `Comparator<TextAnnotation>` **Overrides:**: `compare` in class `AnnotationComparator`

---


### compare

```
public PRFResult compare(List<TextAnnotation> goldTaList,
                         List<TextAnnotation> testTaList,
                         SpanComparator spanComparator,
                         ClassMentionComparator classMentionComparator,
                         int maximumComparisonDepth)
```

:   Overridden - note this ignores all extraneous input parameters and simple returns EntityPartsAnnotationComparator.compare(List
    goldTaList, List testTaList)

    :   **Overrides:**: `compare` in class `AnnotationComparator`
    :   **Returns:**

---


### compare

```
public PRFResult compare(List<TextAnnotation> goldTaList,
                         List<TextAnnotation> taList,
                         SpanComparator spanComparator,
                         ClassMentionComparator classMentionComparator)
```

:   Overridden - note this ignores all extraneous input parameters and simple returns EntityPartsAnnotationComparator.compare(List
    goldTaList, List testTaList)

    :   **Overrides:**: `compare` in class `AnnotationComparator`
    :   **Returns:**

---


### compare

```
public int compare(TextAnnotation ta1,
                   TextAnnotation ta2,
                   SpanComparator spanComparator,
                   ClassMentionComparator classMentionComparator,
                   int maximumComparisonDepth)
```

:   Overridden - note this ignores all extraneous input parameters and simple returns EntityPartsAnnotationComparator.compare(TextAnnotation ta1,
    TextAnnotation ta2)

    :   **Overrides:**: `compare` in class `AnnotationComparator`
    :   **Returns:**

---


### compare

```
public int compare(TextAnnotation ta1,
                   TextAnnotation ta2,
                   SpanComparator spanComparator,
                   ClassMentionComparator classMentionComparator)
```

:   Overridden - note this ignores all extraneous input parameters and simple returns EntityPartsAnnotationComparator.compare(TextAnnotation ta1,
    TextAnnotation ta2)

    :   **Overrides:**: `compare` in class `AnnotationComparator`
    :   **Returns:**


---


|  |  |  |
| --- | --- | --- |
| **Overview** \| **Package** \| **Class** \| **Tree** \| **Deprecated** \| **Index** \| **Help** | |  |
| **PREV CLASS**   **NEXT CLASS** | **FRAMES**    **NO FRAMES**     **All Classes** |  |
| SUMMARY: NESTED \| FIELD \| CONSTR \| METHOD | DETAIL: FIELD \| CONSTR \| METHOD |  |


---
